# Supporting Dementia Caregiving With a Mobile Care Ecosystem: Development and Mixed Methods Study

**DOI:** 10.2196/78759

**Published:** 2025-12-30

**Authors:** Chetna Malhotra, Yanzhen Yue, Chandrika Ramakrishnan, Shimoni Shah, Wenda Chen, Philip Yap, Chin Yee Cheong, Irene Teo, Shiou-Liang Wee, Xiangming Lan, Yi Chen, Chee Seng Chong, Xueying Huang, Ivy Chua

**Affiliations:** 1 Lien Centre for Palliative Care, Duke NUS Medical School, 8 College Road, Singapore, 169857, Singapore; 2 Programme in Health Services Research and Population Health, Duke NUS Medical School, 8 College Road, Singapore, 169857, Singapore, 65 65165692; 3 Institute of High Performance Computing, Agency for Science, Technology and Research, Singapore, Singapore; 4 Department of Geriatric Medicine, Khoo Teck Puat Hospital, Singapore, Singapore; 5 SR Nathan School of Human Development, Singapore University of Social Sciences, Singapore, Singapore

**Keywords:** dementia, caregiving, user-centered design, multicomponent, mobile app, usability, acceptability, artificial intelligence chatbot, AI chatbot

## Abstract

**Background:**

Dementia presents substantial challenges for informal caregivers. A gap remains in technology-driven personalized support tailored to caregivers’ needs.

**Objective:**

This study aimed to develop a theory-driven, multicomponent mobile app specifically designed for caregivers of individuals with dementia and test its usability among end users.

**Methods:**

We developed *CareBuddy*, a mobile care ecosystem based on the stress process model and user-centered design. The app includes personalized assessments and tailored solutions, an artificial intelligence–driven chatbot, GPS-based location monitoring, peer support, a helpline, telemedicine, health care provider integration, and caregiver self-care resources. Development was informed by interviews with caregivers and stakeholders, followed by a 2-phase pilot test involving 18 and 10 participants, respectively, to assess usability and acceptability.

**Results:**

In phase 1, the mean system usability scores increased from 65.4 (SD 11.8) in round 1 to 73.8 (SD 15.9) in round 3, exceeding the benchmark of 68. In phase 2, caregivers rated the app highly, with an overall mean score of 95.4 (SD 8.5) on the Mobile Health App Usability Questionnaire. The domains of ease of use (mean 24.1, SD 2.9), user interface and satisfaction (mean 40.3, SD 3.4), and usefulness (mean 31, SD 3.9) received high Mobile Health App Usability Questionnaire ratings. Participants valued the content focused on dementia management and caregiver well-being. Caregivers appreciated the interactive features: social networking portal, service directory, and conversational large language model. Feedback highlighted areas for improvement, including reducing textual overload and addressing navigational challenges.

**Conclusions:**

*CareBuddy* offers a multifaceted digital solution for dementia caregivers, with high usability and satisfaction. An ongoing trial is evaluating the app’s effectiveness in improving caregiver outcomes.

## Introduction

Dementia is an increasingly pressing public health issue, driven not only by the rising number of diagnoses in aging populations [1] but also by the substantial burden it places on informal caregivers. Informal caregivers, typically family members or close friends, often dedicate an average of 5 hours per day to caregiving tasks, which range from personal care and managing daily routines to ensuring the safety and well-being of individuals living with dementia [2-4]. Informal caregiving is so integral to dementia care that it accounts for more than half of the total costs associated with dementia care [5], underscoring the critical role these caregivers play within the health care system. However, this immense responsibility comes at a high cost to caregivers, affecting their physical, psychological, and emotional well-being and contributing to loss of labor market productivity [6-12].

To address these challenges, caregivers require multifaceted support, including timely and accurate information, emotional and social support, and access to health care providers [13-15]. Meeting these diverse needs requires comprehensive, multicomponent interventions that can provide not only sustained, holistic care but also immediate assistance when required.

Technology-based platforms have emerged as a promising avenue for supporting dementia caregivers. Systematic reviews of technology-driven interventions have shown that caregivers highly value certain key elements, such as peer support, direct contact with health care providers, and information tailored to their specific needs, rather than generalized content [16-20]. Psychological support is also considered crucial in these interventions [21]. These features are most effective when integrated as multicomponent interventions.

Mobile health apps offer an accessible and convenient platform. However, 2 scoping reviews found that most existing apps, including those commercially available, focused on monitoring, tracking, task management, mental health support, health education, and caregiver communication but fell short of meeting caregivers’ needs [22,23]. A systematic review identified only 6 dementia caregiver apps that have undergone research evaluation, most of which provided task-based support [24]. Current apps thus remain limited, often lacking high-quality tailored content, interactivity, personalization, and theoretical grounding in their development [25-27].

A mobile app that is multicomponent, theory driven, and personalized to caregivers’ unique needs could provide both sustained support and real-time assistance and serve as a one-stop solution for caregivers, creating an integrated caregiving ecosystem. The effectiveness of such an app would depend on several critical factors, including the app’s design, user-friendliness, and the quality of support it offers.

We therefore developed *CareBuddy*, a theory-driven, multicomponent mobile app specifically designed for caregivers of individuals with dementia. The app focuses on enhancing sustained and ongoing support, ensuring continuity of care, and fostering a sustainable caregiving environment. This paper aims to describe the development process of the “*CareBuddy*” app and its multicomponent features and to present the results for its usability and acceptability testing among end users.

## Methods

### Overview

We used user-centered design principles and iterative processes to develop the *CareBuddy* app [28,29]. We reported the study in accordance with the COREQ (Consolidated Criteria for Reporting of Qualitative Studies) [30] (Checklist 1).

### Ethical Considerations

The study was approved by the National University of Singapore Institutional Review Board (NUS-IRB-2022‐309). All participants signed a written informed consent before participation. We assigned subject codes for participants, and the data were deidentified to ensure anonymity of responses. Participants received S$30 to S$70 (S$1=US $0.77) depending on the phase of the study.

### Development Process

#### Assessment of User Requirements

We conducted qualitative in-depth interviews with family caregivers of people with dementia, policymakers involved in planning dementia programs, clinicians, and community dementia service providers to identify desired features for a mobile app designed for caregivers of individuals with dementia. Using purposive sampling, we recruited family caregivers through referrals from community dementia organizations and an ongoing study, whereas policymakers, clinicians, and service providers were approached through key contacts in relevant organizations. A trained qualitative researcher conducted interviews, which were audio recorded, transcribed, and analyzed on NVivo 11 (Lumivero) through content analysis.

Two informal caregivers of individuals with dementia guided the research team on the design and content of the *CareBuddy* app. Study team members met with them frequently to elicit feedback.

#### App Features

**Figure 1.**
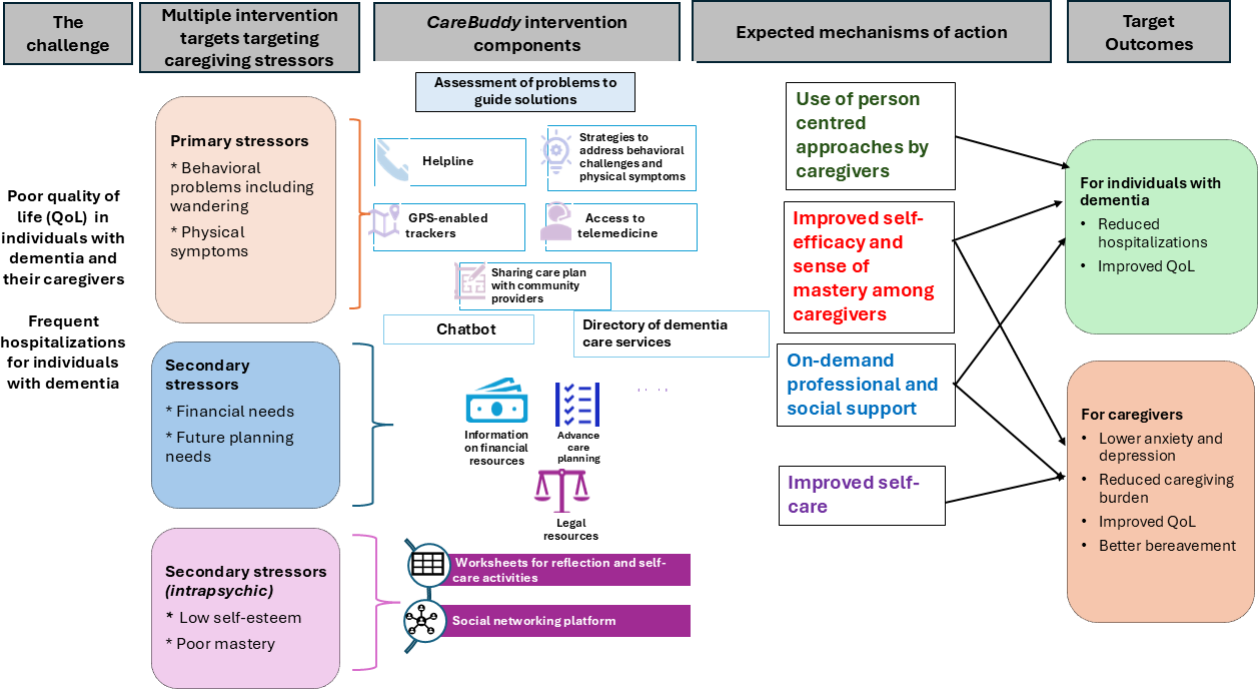
Logic model for development of the *CareBuddy* mobile care ecosystem.

Guided by the stress process model [31], we developed a logic model to develop the “*CareBuddy*” app (Figure 1). The stress process model, an established framework, describes the relationship between caregiving stressors, psychosocial resources, and caregivers’ psychosocial well-being [31,32]. The framework thus provides a foundation to develop an integrated caregiving ecosystem that addresses caregiving stressors and delivers comprehensive support by enhancing caregivers’ access to tailored resources, coping strategies, and social support networks.

### Pilot Testing

#### Overview

We conducted pilot testing in 2 phases using a mixed-method design incorporating both qualitative feedback and quantitative assessments for app usability.

#### Phase 1

In this phase, we aimed to gather feedback on the app’s content and functionality. Participants interacted with the prototype version of the app using the interviewer’s smartphone. The interviewer then guided the participant through the app’s various components using a topic guide to prompt open-ended discussions. This phase involved 3 rounds of in-depth interviews, with the app iteratively updated after each round based on participants’ feedback. The interview guide, developed by the study team, explored key aspects of user experience, app functionality, the relevance of the app’s solutions for managing behavioral and physical symptoms of individuals with dementia, perceived usefulness, and suggestions for improvement. We also collected demographic data and assessed usability using the 10-item System Usability Scale (SUS) [33].

We purposively sampled family caregivers and health care providers. Eligible caregivers were Singaporean or permanent residents aged ≥21 years, involved in making treatment decisions for a family member with dementia, had been providing care for at least 6 months and were planning to do so for the next 2 months, able to read and write English, and had intact cognition as determined through the Abbreviated Mental Test (only for those aged ≥65 y). Health care providers included those providing health or social care within community organizations or hospitals. The interviews were conducted in English by a trained researcher with no prior relationship with the participants. Sessions were held at a time and place convenient for the participants, lasting 1 to 2 hours.

#### Phase 2

This phase assessed caregivers’ app engagement, usability dimensions including satisfaction and acceptability, and subjective experiences. Caregivers downloaded the app (iOS or Android version) onto their own mobile phones, with a researcher providing an orientation on its features and navigation. If the care recipient was mobile, caregivers additionally received a GPS tracker for tracking their location.

Eligibility criteria for family caregivers remained the same as in phase 1, with the additional requirement of owning an Android or iOS mobile phone. Caregivers of individuals residing in long-term care facilities were excluded.

Following informed consent, we collected demographic data for the caregivers (eg, age, gender, relationship, and education) and the individual with dementia (eg, age and gender) through a survey. At the end of the 1-month trial, app usage was tracked throughout the month, including average engagement time per week, time spent on each component, and frequently used components. We administered the Mobile Health App Usability Questionnaire (MAUQ), English stand-alone patient version, for the intended audience, family caregivers of individuals with dementia [34]. MAUQ, a validated 18-item questionnaire, assesses usability in 3 dimensions (ie, ease of use, interface and satisfaction, and usefulness) on a 7-point Likert scale, ranging from 1 (strongly disagree) to 7 (strongly agree) [34]. Caregivers also participated in open-ended interviews exploring their experiences and subjective feedback on the relevance and perceived usefulness of the app. The open-ended portion was audio recorded and transcribed. Interviews lasted 10 to 25 minutes.

### Analysis

#### Quantitative

We summarized demographics and calculated SUS scores both overall and by interview round to assess the app’s usability evolution. For the phase 1 pilot, we compared the SUS scores to the benchmark mean of 68, which indicates good usability [35,36]. In the phase 2 pilot, we calculated total and average scores assessing MAUQ usability dimensions, that is, ease of use, interface and satisfaction, and usefulness, including satisfaction and acceptability, with higher total and average scores indicating greater usability [34]. An average score lower than 4 meant poor usability [37]. We also summarized app usage data in terms of the number of hours used, components used, and time spent on each component in phase 2.

#### Qualitative

Using NVivo 11 software, 2 team members independently coded transcripts and conducted content analysis [38]. We developed a code book based on the User Version of the Mobile Application Rating Scale [39], categorizing feedback into key domains: engagement, functionality, aesthetics, information quality and quantity, and subjective feedback. We synthesized user feedback within these domains and incorporated suggestions for refining the app.

We triangulated quantitative and qualitative findings on usability and engagement.

## Results

### Development Process

#### Assessment of User Requirements

##### Participant Demographics

We interviewed 22 participants (ie, 11 caregivers, 6 community dementia providers, 3 clinicians, and 2 policymakers), with a mean age of 49 (SD 10.4) years, 73% (n=16) were women, and half (n=11, 50%) were caregivers of individuals with dementia (details in Table 1).

We grouped participants’ desired features into three categories: education, communication, and listing/directory.

**Table 1.** Participant characteristics.

Item	Assessment of user requirements		Pilot: phase 1		Pilot: phase 2, caregivers (n=10)
	Caregivers (n=11)	Noncaregivers (n=11)	Caregivers (n=11)	Health care providers (n=7)	
Age (y), mean (SD)	53.7 (9.6)	44 (8.9)	58.5 (9.5)	38.9 (11)	58.5 (9.5)
Gender, n (%)					
Male	4 (36.4)	2 (18.2)	4 (36.4)	4 (57.1)	2 (20)
Female	7 (63.6)	9 (81.8)	7 (63.6)	3 (42.9)	8 (80)
Ethnicity, n (%)					
Chinese	9 (81.8)	—^a^	11 (100)	5 (71.4)	9 (90)
Non-Chinese	2 (18.2)	—	0 (0)	2 (28.6)	1 (10)
Primary language, n (%)					
English	—	—	9 (81.8)	7 (100)	—
Chinese	—	—	2 (18.2)	0 (0)	—
Marital status, n (%)					
Married	—	—	10 (90.9)	—	5 (50)
Never married	—	—	1 (9.1)	—	3 (30)
Divorced	—	—	0 (0)	—	2 (20)
Widowed	—	—	0 (0)	—	0 (0)
Education, n (%)					
No formal education	0 (0)	—	0 (0)	—	0 (0)
Secondary	3 (27.3)	—	3 (27.3)	—	1 (10)
Junior college/polytechnic/diploma	3 (27.3)	—	3 (27.3)	—	3 (30)
University and above	5 (45.5)	—	5 (45.5)	—	6 (60)
Relationship with person with dementia, n (%)					
Spouse	1 (9.1)	—	5 (45.5)	—	4 (40)
Children	9 (81.8)	—	5 (45.5)	—	2 (20)
Sibling	0 (0)	—	1 (9.1)	—	1 (10)
Others	1 (9.1)	—	0 (0)	—	3 (30)

a Not applicable because data not collected.

##### Education

Participants desired short videos for caregivers focusing on daily living skills and management of troublesome behaviors among individuals with dementia. They also expressed interest in newsletters, articles, and support group information. Additional requests included information on financial and legal considerations, self-care strategies to address caregiver stress, and self-assessment tools to personalize support. A guide on how to navigate the app was also suggested.

##### Listing/Directory

Participants wanted directories for various services, including daycare centers, respite care services, dementia-specific community services, and telemedicine providers.

Summary of desired app features with illustrative quotes is detailed in Table S1 in Multimedia Appendix 1.

### App Features

We describe the *CareBuddy* app’s features under three aspects: app components, user interface design, and system features.

### App Components

#### Overview

On the basis of the user requirements and the stress process framework–based logic model, we developed app components to address both primary and secondary stressors (Figure 1).

#### Addressing Primary Stressors of Dementia Caregiving

The app targets primary caregiving stressors such as behavioral symptoms (eg, aggression, agitation, mood swings, hallucinations, disorientation, and refusal of care) and physical challenges (eg, mobility issues, eating difficulties, dry skin, toileting problems, communication difficulties, and sleep disturbances). Upon logging in, caregivers are guided through an assessment using a series of questions to identify current behavioral and physical symptoms, as well as their potential underlying causes. On this input, the app offers tailored, practical management strategies. Specifically, for the care recipient’s behaviors, the approach uses the unmet needs model (which views behaviors as expressions of unarticulated needs), the progressively lowered stress threshold model (which emphasizes the reduced capacity of the care recipient to deal with stress or stimuli), and the ABC (antecedent, behavior, and consequences) models. The solutions are further derived using the DICE (describe, investigate, create, evaluate) and P.I.E.C.E.S. (Physical. Intellectual. Emotional. Capabilities. Environment. Social.) frameworks [40,41]. The content was developed in consultation with dementia care providers and from existing resources published globally. All content was reviewed and validated by 2 geriatricians on the study team with expertise in dementia care.

A particularly difficult behavioral symptom, wandering, is addressed through a GPS location monitoring function integrated into the app. This feature allows caregivers to monitor the real-time location of their care recipients using GPS-enabled trackers. Caregivers can view the last detected location of their care recipients directly through Google Maps. Additionally, the app provides step-by-step navigation to the tracked location. The app monitors the GPS tracker’s battery level and provides reminders to recharge the battery.

To address primary stressors, *CareBuddy* also facilitates collaborative care management through a care plan function, which supports information sharing between family caregivers and health care professionals. The Care Plan includes a section on the individual’s hobbies and interests to promote person-centered care. Caregivers can also use a centralized messaging tool to share updates and discuss care plans with providers. The app also allows caregivers to apply for care center transfers, with approvals managed by health care providers. A secure QR code–based system enables multiple caregivers (eg, family members or friends) to coordinate care efficiently. By streamlining communication and care planning, *CareBuddy* strengthens coordinated, person-centered care.

To ensure timely access to health services, *CareBuddy* integrates telemedicine access via deep links to third-party provider apps. Users can initiate virtual consultations with established general practitioners directly through *CareBuddy*, eliminating the need to search for separate telemedicine apps. This integration enhances continuity within *CareBuddy’s* digital ecosystem. Instructional content is also provided to guide caregivers on using these services effectively.

In moments of acute distress, caregivers can also access a crisis support helpline within the app, connecting them directly to trained social workers who provide emotional support, practical guidance, and crisis intervention.

A large language model (LLM)–powered chatbot is integrated into the app to provide personalized, real-time responses to caregivers’ queries by offering advice, summarizing key strategies, and encouraging engagement with relevant app components. This chatbot uses GPT-4 as the default language model for generating responses and GPT-3.5 for contextual analysis based on the knowledge database. The system is built on a retrieval-augmented generation architecture, where relevant knowledge items are retrieved from the database and used as context in prompts to the language model. This enables the chatbot to generate responses that are both contextually accurate and conversationally coherent. The chatbot integrates both an internal knowledge repository and external knowledge sources to generate accurate, contextually relevant responses. The internal repository comprises a structured question-and-answer (Q&A) database, curated by the study team to address key caregiving stressors. To enhance response accuracy, we implemented natural language processing to semantically map user queries to the most relevant Q&A entries, ensuring precise information retrieval. A tiered, confidence-based retrieval system further optimizes response generation, with confidence scores derived from cosine similarity between user queries and knowledge-based questions. Queries scoring above 95% are directly matched to Q&A entries with LLMs adding empathetic context, scores between 90% and 95% synthesize information across multiple sources, and scores below 90% engage ChatGPT with domain-specific context for broader, tailored responses. These thresholds were empirically determined through user testing to balance accuracy for specific queries with generalization for broader or ambiguous ones. Testing confirmed that high-confidence matches yield precise answers, whereas lower-confidence matches generate broader, contextually relevant summaries. To ensure safety, the chatbot responds strictly within its knowledge scope at high confidence and, in urgent or sensitive cases, directs users to immediate resources such as helplines rather than attempting diagnostic or therapeutic advice. Personal information is coded, protected, and delinked from individual users, ensuring all chatbot conversations are completely anonymous.

#### Addressing Secondary Stressors of Dementia Caregiving

To address secondary stressors such as financial burden and future care planning, the app offers curated content on financial resources and subsidies, along with guidance on how to do advance care planning, power of attorney, making a will, and Central Provident Fund nominations. Caregivers also receive information on the anticipatory trajectory of illness for dementia so that they can plan for the evolving needs of their care recipient as the condition progresses.

To reduce internal caregiver stress and improve emotional well-being, *CareBuddy* includes a dedicated self-care module featuring psychologist-developed worksheets. These resources help caregivers reflect on their caregiving identity and journey, recognize signs of burnout, engage in self-care, and build coping strategies for anticipatory grief and emotional resilience.

The app also provides access to the user’s phone calendar, which allows caregivers to create personalized event reminders. This function helps users keep track of important dates, appointments, or caregiving tasks and provides timely alerts to ensure they receive the necessary prompts to take action. This feature responds directly to caregiver feedback and is designed to support daily organization.

Finally, *CareBuddy* facilitates peer support through an in-app online forum, available both on the app and via a web portal. This space allows caregivers to seek help, share experiences, offer support, get connected with other caregivers, and build community. Users can flag inappropriate posts, block others if needed, and benefit from a backend “Censor Curse Words” function that ensures respectful communication. An LLM-based artificial intelligence (AI) bot named “Sparky” is available in the online forum to stimulate user interactions and facilitate engagement with caregivers. The AI bot “Sparky” generates initial replies to caregivers’ new posts and provides comments when requested by caregivers.

### User Interface Design

#### Caregiver Interface

The *CareBuddy* app interface is intentionally designed to be user-friendly, with frequently used functions placed in prominent positions and enhanced with high-resolution icons. A text-to-speech feature allows users to listen to information instead of reading, supporting accessibility. Onboarding instructions and reminders are integrated to support user navigation and regular engagement.

#### Health Care Provider Interface

The *CareBuddy* caregiver app is connected to a dedicated health care provider version that supports them in collaborating with family caregivers. Within the health care provider version interface, users can approve new clients with dementia, update client information, and engage in care planning discussions with caregivers. The health care provider version also enables providers to participate in the online forum, exchanging insights and offering support to caregivers and other stakeholders.

#### Moderator Interface

The *CareBuddy* caregiver app is also connected to a moderator version. Moderators of the online forum can manage the forum via the *CareBuddy* web portal, with tools to pin or feature posts and share multimedia caregiving resources.

### System Features

### Overview

From a system development perspective, we implemented a scalable hybrid architecture combining single-service and multiservice systems to efficiently manage traffic capacity and enhance system resilience. Information entered by caregivers is stored securely in the cloud database and synchronized across services to allow continuity across sessions.

#### System Usage Tracking

*CareBuddy* includes an embedded system usage log that captures detailed user activity across the app. This feature tracks user actions to better understand caregiver needs and app engagement patterns. In addition, a data-capturing component tracks interactions within the online forum, including posts, threads, and social dynamics among users. These built-in analytics features support future improvements and enable ongoing research into user behavior.

#### System and Messaging Notifications

*CareBuddy* integrates a comprehensive notification system using Firebase Cloud Messaging to keep users engaged and informed. The app supports system-scheduled notifications that provide timely nudges for user engagement and updates within the *CareBuddy* environment. In-app notifications are triggered by specific user interactions, such as messaging between family caregivers and health care professionals, ensuring users stay informed of meaningful exchanges and collaborative care efforts. Additionally, push notifications are delivered even when the app is closed, allowing critical information, such as new messages, care plan updates, or forum interactions, to reach users without delay. These notifications are customized based on user activity and context, including upcoming appointments or scheduled reminders.

#### Chat History Management

This feature was designed to support comprehensive storage, retrieval, and analysis of past user interactions, incorporating user metadata, conversation sessions, and analytical reporting to continuously refine chatbot performance and personalization.

#### Data Privacy and Security

Data privacy and security measures were implemented for proper handling of sensitive features and data within the app. GPS location data and chatbot records are stored in an anonymized format and not linked to personally identifiable information in the backend database. Thus, data are decoupled from patients’ profile data, and identifiable information is separately stored and managed by the research team.

Before the *CareBuddy* mobile app was made available for download (on both the Apple App Store and Google Play Store), the app was reviewed by both platforms. *CareBuddy’s* adherence to best practice measures ensured it satisfied both platforms’ prevailing compliance and regulatory requirements and ensured privacy and security protection for end users. Furthermore, the privacy policy and End User License Agreement were also made available to end users to elaborate on how content filtering and moderation are implemented to ensure rigor, appropriateness, and security of the app. *CareBuddy* app users also provided their consent and acceptance of the service terms during the account registration process.

Moreover, all user data and interaction records (including data related to sensitive features) are stored securely in the Amazon Web Services cloud, with its access strictly limited to the designated system administrator from the research team and further protected with multifactor authentication for enhanced security. These measures ensured that user privacy, security, and ethical considerations were upheld throughout the development and pilot testing.

**Figure 2.**
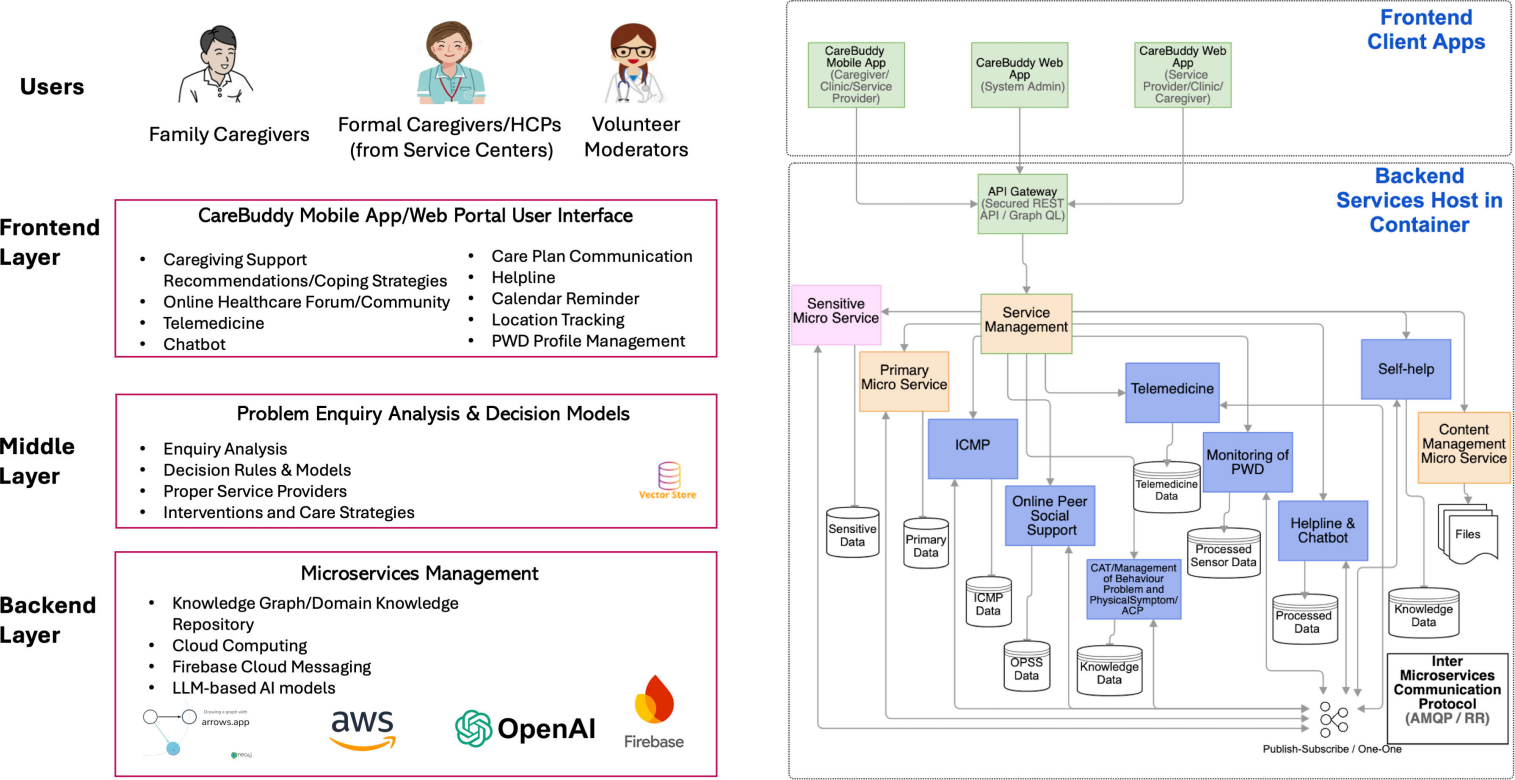
*CareBuddy* system structure. ACP: advance care planning; AI: artificial intelligence; AMQP/RR: advanced message queuing protocol/request response; API: application programming interface; CAT: computerized adaptive testing; HCP: health care professional; ICMP: integrated client management portal; LLM: large language model; OPSS: online peer social support; PWD: person with dementia; REST API: representational state transfer application programming interface.

The *CareBuddy* system structure is presented in Figure 2, and sample screenshots of the components, interface, and features are presented in Figure S1 in Multimedia Appendix 1.

### Pilot Testing

A total of 28 participants were involved in the pilot testing, with 18 individuals (11 caregivers and 7 health care providers) in phase 1 and 10 caregivers in phase 2. This sampling aligns with the general rule of thumb, which suggests that 80% of all potential usability problems can be identified by testing with 5 to 10 users [42]. Participant characteristics are described in Table 1.

#### Quantitative

##### Usability Scores in Phases 1 and 2

Usability of the app improved in rounds 2 and 3 of phase 1. The overall mean usability scores increased from 65.4 (SD 11.8, range 47.5-80) in round 1 to 77.5 (SD 7.2, range 70-87.5) in round 2 and 73.8 (SD 15.9, range 52.5‐97.5) in round 3. Notably, rounds 2 and 3 exceeded the benchmark usability score of 68 (Table 2). Overall, 67% (12/18) of participants reported SUS scores above 68, including 73% (8/11) of caregivers and 57% (4/7) of health care providers.

**Figure 3.**
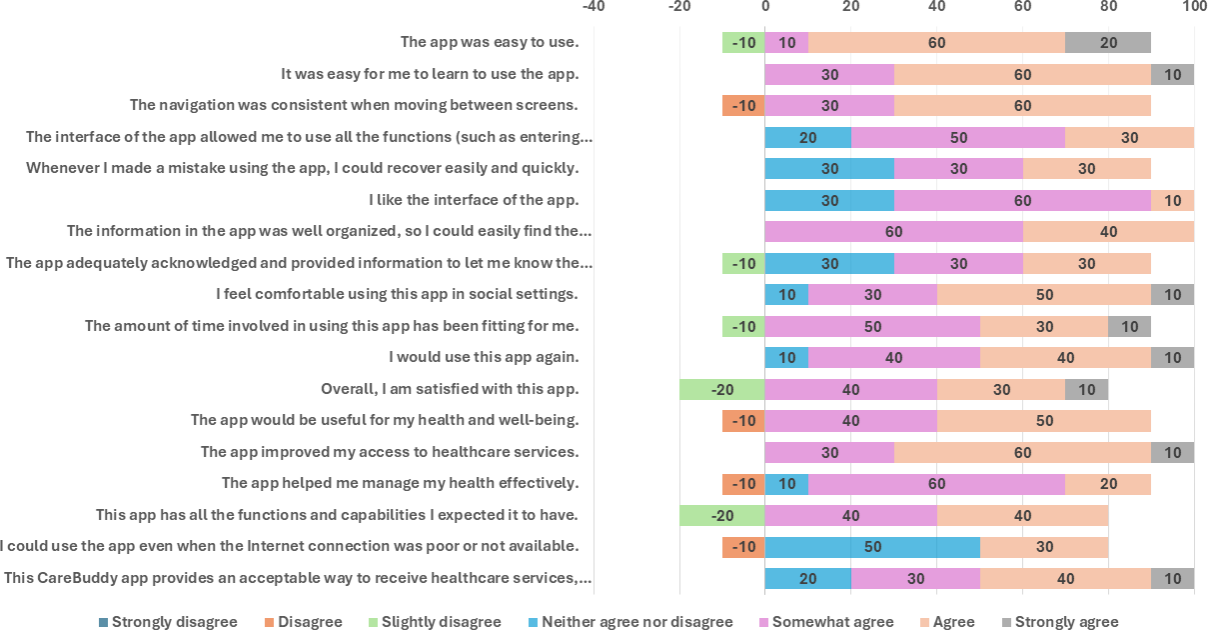
Usability and satisfaction—Mobile Health App Usability Questionnaire results.

In phase 2, participants rated the app highly, with an overall total mean score of 95.4 (SD 8.5). The ease of use (mean 24.1, SD 2.9), user interface and satisfaction (mean 40.3, SD 3.4), and usefulness (mean 31, SD 3.9) were high (Table 3). Figure 3 shows that the majority of the participants somewhat agreed, agreed, or strongly agreed with all items of MAUQ. All participants either agreed or strongly agreed with “Overall, I am satisfied with this app.”

**Table 2.** System Usability Scale scores by participant group (phase 1).

	Caregivers (n=11)	Health care providers (n=7)	Overall (n=18)
Round 1 scores, n	4	2	6
Range	47.5‐80	57.5‐67.5	47.5‐80
Mean (SD)	66.9 (14.3)	62.5 (7.1)	65.4 (11.8)
Round 2 scores, n	4	2	6
Range	70‐87.5	72.5‐85	70‐87.5
Mean (SD)	76.9 (7.7)	78.8 (8.8)	77.5 (7.2)
Round 3 scores, n	3	3	6
Range	52.5‐97.5	62.5‐72.5	52.5‐97.5
Mean (SD)	78.3 (23.2)	69.2 (5.8)	73.8 (15.9)

**Table 3.** Usability and satisfaction score among caregivers (n=10; phase 2).

Mobile Health App Usability Questionnaire domains	Sample score range	Score, mean (SD)
Ease of use (range 5-35)	18‐30	24.1 (2.9)
Interface and satisfaction (range 7-49)	34‐47	40.3 (3.4)
Usefulness (range 6-42)	23‐35	31 (3.9)
Overall (range 18-126)	79‐107	95.4 (8.5)

##### App Usage in Phase 2

The backend system usage tracking data showed that informational resources, which included content on understanding dementia and managing behavioral and physical symptoms, financial assistance, and future planning, and the directory of service providers, were used for a median of 18.8 minutes, with a maximum of 35.5 minutes. Resources focused on caregiver self-care, such as managing stress, caring for themselves, navigating the caregiving journey, and coping with grief, were used for a median of 7.5 minutes with a maximum of 28.5 minutes, and the online social forum for 9.4 minutes with a maximum of 1.7 hours.

### Qualitative

#### Overview

The analysis of substantive interview feedback and suggestions from phases 1 and 2 is summarized under 5 domains of the User Version of the Mobile Application Rating Scale, with representative quotes from participants.

#### Domain: Information and Content

Participants in phase 1 found the information credible, high quality, and easy to understand, covering relevant caregiving topics. Users in round 1 found the text wordy, suggesting bullet points, visuals, and a ‘view more’ option for details. With progressive iterations and changes made in response to suggestions from earlier rounds, most participants in round 3 were satisfied with content clarity and organization. They perceived the content to be rich, straightforward, containing extensive resources, and providing solutions for managing common behavioral symptoms. One health care provider mentioned:

*Based on the three different broad categories I have clicked, I think so far, I'm quite impressed. I think that it gives me a quick glimpse of you know why my loved one may exhibit the some behaviour. And then you don't just leave me hanging, you also give me certain solution to work towards*.

[Phase 1, participant 16]

Caregivers in phase 2 valued the content on dementia trajectory, management of behavioral and physical symptoms, predeath grief, stress management, and self-care content, finding it relatable. They found the content well organized and easy to use. In comparison to existing local apps, they found *CareBuddy* comprehensive and eliminated the need for search engines such as Google.

#### Domain: Usability and Functionality

While most participants in phase 1 found the app easy to use, some struggled with navigation and chatbot performance. Participants suggested making the chatbot responses more empathetic. In response, we enhanced navigation by adding subheaders for each section and incorporating hyperlinks to external resources. We also improved the chatbot’s vocabulary, ensuring sensitivity for better responsiveness. A health care provider noted:

*It is pretty much comprehensive and easy to navigate. These are the common things that most of them [caregivers] will be able to look at*.

[Phase 1, participant 09]

In the 1 month of using the app in phase 2, caregivers found the search function in the directory intuitive. They also used the interactive app elements such as a GPS tracker, worksheets, a chatbot, and a directory extensively. Most participants found no issues with navigation; however, some reported missing links to external sites and noted that the tracker function required frequent refreshing to update the care recipient’s location.

#### Domain: Aesthetics and Design

Early feedback from phase 1 cited small fonts, cluttered layout, and dull colors. In response, we made adjustments to text size, formatting, and color contrast for improved readability. We added an audio option for users who preferred listening to the content. Later feedback was very positive. A health care provider reported:

*I like that you colour coded into the broad category so that, I can instinctively try to find a common theme and then go to it directly. So yeah, I thought that was very nice*.

[Phase 1, participant 16, HCP]

#### Domain: Engagement

Many participants in phase 1 showed interest and eagerness to read the app content and suggested providing empathetic responses to ensure appropriateness. Moreover, providing practical solutions rather than theoretical responses ensured engagement with the app.

In phase 2, at least 40% of caregivers used the app every day. Many participants had positive feedback and felt happy to connect with fellow caregivers on the community portal. Others preferred to just hear others and found information shared by other caregivers to be helpful. They reported that the moderators did a good job of initiating discussions and engaging users. One caregiver reported:

*In the beginning I was [checking] almost every day. Not only every day like different times of the day*. *...I think the moderator did quite good job in like throwing questions like what the activities are you do and then I shared. The last one he asked is what do you do at the social club? It’s good to share my knowledge. So, I think that is one of the most useful one (component), the one that I go to a lot*.

[Phase 2, participant 01]

#### Domain: Relevance and Perceived Usefulness

Feedback from participants in early phase 1 was mixed. Some felt the app to be more relevant for early dementia diagnosis, whereas others believed it was suited for later-stage dementia care. In response, we enhanced the content of the app, incorporating more examples of how care can be provided. Caregivers in round 3 perceived the app to be more useful, functioning as a valuable guide and offering real-time support with practical solutions for addressing behavioral and physical symptoms. Some of the participants were keen and excited about the launch of the app due to the lack of a comprehensive app such as *CareBuddy*. A caregiver noted that:

*I think it’s very helpful to care partner like me. When I need help, I know this app is around. When first diagnosed, I was not too sure what to do. Totally lost, feel so scary, sad...upset, worrying, yeah. So, it’s like, you know, very helpless, do not know where to go*.

[Phase 1, participant 14]

A health care provider also reported that:

*I am very excited for this app to be launched because right now in the market don’t think there is really an app which covers so much information.*

[Phase 1, participant 16]

Over a 1-month trial, caregivers found the app solutions helpful in managing behavioral challenges (eg, agitation). The easy access to resources and content enabled a better understanding of dementia progression. Caregivers reported that the curated content saved time, providing quick access to care resources such as nursing homes, respite care, and mental health helplines:

*I saw the list and I found the ones [service providers] that are nearer to my house. And of course, I'm very happy with the phone numbers. Because very important to have phone numbers I can call immediately*.

[Phase 2, participant 06]

Participants appreciated the *CareBuddy* as comprehensive and were keen to recommend the app to others. One of the caregivers reminisced that having the *CareBuddy* app would have been immensely valuable when their loved one had been newly diagnosed with dementia.

The changes made to the app based on participants’ suggestions by phases are described in Table S2 in Multimedia Appendix 1, and qualitative analysis with illustrative quotes is detailed in Multimedia Appendix 2.

## Discussion

### Principal Findings

*CareBuddy* is a theory-informed mobile intervention purpose-built to support the complex and evolving needs of dementia caregivers. Co-developed by a multidisciplinary team, including health care providers, computer scientists, health services researchers, and caregivers themselves, *CareBuddy* delivers an integrated digital ecosystem that empowers caregivers across the care journey by addressing the practical, emotional, and logistical challenges of caregiving. The app provides continuous, on-demand support through access to educational resources, peer and professional communication channels, and immediate crisis assistance via a built-in helpline and telemedicine access. A mixed methods pilot study demonstrated high usability and satisfaction with user feedback guiding iterative refinements to enhance *CareBuddy*. By offering timely, personalized, and scalable support, *CareBuddy* shows strong potential to improve caregiver well-being, reduce burden, and promote sustainable, high-quality home care for individuals living with dementia.

In comparison to existing researched apps for caregivers of persons with dementia that have limited features (eg, tracking and caregiver self-care component) [24], *CareBuddy* is grounded in the stress process model and targets caregiving stressors through a conversational AI for caregiver queries, a helpline, telemedicine services, and a moderated social networking portal providing real-time assistance. Additional components on financial and legal information, stress-management exercises, self-care resources, and a directory of dementia care services target secondary stressors supporting long-term caregiver well-being. An interactive care management portal, though not tested in this study, connects caregivers to service providers. Its co-design process, involving caregiver representatives, ensured alignment with real-world needs. Features were informed by interviews with caregivers and health care professionals, ensuring practical utility. Thereafter, an iterative mixed methods design further refined the app based on user input. The app is available on both iOS and Android.

Quantitative usability testing showed strong results. The mean (SUS) scores exceeded the threshold of 68, with 67% of participants scoring above this benchmark. The MAUQ scores indicated high overall satisfaction, with strong ratings for ease of use, user interface, and usefulness (overall mean score 95.4, SD 8.5). Caregivers rated satisfaction statements positively, reinforcing *CareBuddy’s* acceptability and usability.

Notably, *CareBuddy* is designed to be a dynamic tool, as new features are continuously being developed and tested, with plans for phased integration into future versions of the app. These include decision aids to help caregivers choose between comfort feeding or tube feeding and enhanced AI-driven support for the online platform.

### Future Implications

Importantly, the app is built on an adaptable and modular architecture, allowing for seamless customization to suit different health care systems, cultural contexts, and caregiving environments. Whether implemented in community-based care settings, acute hospitals, or long-term care facilities, the *CareBuddy* platform can be configured to align with local workflows, regulatory requirements, and user preferences. This flexibility ensures that *CareBuddy* is not only scalable but also sustainable across diverse care ecosystems.

### Limitations

Despite its promise, this study has limitations. The pilot involved a small sample size and short follow-up duration, consistent with similar studies [43]. While usability and acceptance testing have been essential, further research is needed to assess caregiver engagement and the app’s impact on caregiving outcomes. A 2-arm hybrid type 1 randomized controlled trial is planned to evaluate implementation and effectiveness. Additionally, long-term user retention remains a challenge, as prior studies indicate sharp declines in engagement after initial download [44,45]. To mitigate this, we have incorporated a notification system to encourage sustained use and maximize the app’s impact. New additional features are being tested and will be added to the app, making the app dynamic. Finally, the app is designed to be adaptable and modular, that is, it can be adapted to different contexts.

### Conclusions

*CareBuddy*, envisioned as a comprehensive digital caregiving hub, holds promise to address the multifaceted challenges of dementia caregiving. Its co-design methodology and iterative feedback loops have ensured that the app is closely aligned with the real-world needs of dementia caregivers, enhancing its usability, accessibility, and relevance. Future research, including a randomized controlled trial, will evaluate the app’s long-term effects on caregiver outcomes and key implementation metrics, such as engagement, acceptability, and sustainability. Concurrently, plans are in place to integrate *CareBuddy* into routine care delivery, with adaptations to accommodate different health care systems, languages, and cultural contexts. These efforts aim to extend the app’s relevance and scalability across diverse international settings, broadening its reach and impact on dementia caregiving worldwide.

## Supplementary material

10.2196/78759 Multimedia Appendix 1 Qualitative themes on desired app features with illustrative quotes, updates made to the CareBuddy app across interview rounds, and user-interface screenshots.

10.2196/78759 Multimedia Appendix 2 Qualitative analysis with illustrative quotes.

10.2196/78759 Checklist 1 COREQ checklist.

## Abbreviations

ABC: antecedent, behavior, and consequences
AI: artificial intelligence
COREQ: Consolidated Criteria for Reporting of Qualitative Studies
DICE: describe, investigate, create, evaluate
LLM: large language model
MAUQ: Mobile Health App Usability Questionnaire
P.I.E.C.E.S.: Physical. Intellectual. Emotional. Capabilities. Environment. Social.
Q&A: question and answer
SUS: System Usability Scale
