# Supplementary material for: Supporting Dementia Caregiving With a Mobile Care Ecosystem: Development and Mixed Methods Study
Source: JMIR Aging. 2025 Dec 30;8:e78759. doi: 10.2196/78759 (PMC12810113; doi:10.2196/78759)

**Supplementary Appendix 1: Tables and figures**

**Table S1: Qualitative findings on desired app features with relevant quotes**

| **Topics** | **Areas** | **Sample quotes** |
| --- | --- | --- |
| **User interface** | User friendly | *Usability is important. It needs to be user friendly.* NA20_CG |
|  | Easy navigation | *I would hope that the app is arranged in a way that it is easy and self-explanatory* *because quite a few apps that I’ve been to, have to bash around before actually find the right section that I want to go to. So it's got to be clearly labelled, subject specific*. NA14_CG |
| **Desired app components** | Education | *How to handle dementia patient. Very good if there is some video to show how to handle them. NA22_CG*  *Like every few months it can pass on some magazine newsletter kind of thing. So, might be good to know if there is some talk or some tips or takeaway can can-can be on that too. NA09_CG*  *Reduce our stress, how to handle the stress. It can show video on how to manage stress. NA22_CG* |
|  | Communication | *I am reaching a lot of stress, I need—I-I need help. I call SOS hotline. helpline for services like—where I can find a solution NA07_CG*  *Useful to connect with other spouses and like-minded people…Maybe someone that they can talk to, like teleconference doctors, someone that they can ask (questions)* NA05_CG |
|  | Listing/directory | *We are looking for a eldercare centre. it will be good if there is a list of those centre that will accept dementia patients* NA09_CG |
|  | Chat bot | *I like to have a person that I can chat with. Yeah, , so if I can speak to a bot and then a bot can transfer me to a live uh person to-answer specific questions NA02_CG* |

**Table S2: Changes made to CareBuddy App after subsequent rounds of participant interviews**

| **Changes made** | **Pre-Usability Testing** | **After Phase 1- Round 1** | **After Phase 1-Round 2** | **After Phase 1-Round 3** | **After Phase 2** |
| --- | --- | --- | --- | --- | --- |
| General Changes | **Optimized Text and Layout**: Improved text formatting, reduced length for readability, and clarified instructions to enhance navigation. | **Caregiver-Friendly Language**: Simplified language for better accessibility and understanding. | **Enhanced User Experience**: Added more links and tabs at the bottom of solutions to direct users to other components within the CareBuddy app and embedded all videos in solutions for easier access. | **App Enhancements**: Increased text font size, improved professionalism and aesthetics, and refined account registration, sign-in, and password recovery processes. Modified the full-screen icon for better intuitiveness. | **Improved notification system: Improved app notification message that will be sent periodically to remind users to use the app to aid them in dementia caregiving.** |
|  | **Refined Question and Solution Format**: Replaced Yes/No responses with clickable options to minimize redundancy and reduce user fatigue; reorganized solutions under relevant headings. | **Improved Hyperlinks**: Added functional hyperlinks to solutions and service providers, with text-only links. | **Improved Content and Engagement**: Fixed spelling and grammatical errors, improved image clarity with pop-up windows, and added more videos and hyperlinks to boost engagement. Enhanced content quality with participant feedback and made introductory links more personalized for caregivers' needs. | **New Features**: Introduced a healthcare provider version of the CareBuddy app, added a CarePlan component for caregivers to create profiles and link with healthcare providers, and added an activities section for caregivers to interact with providers and update loved ones’ health status. | **Updated CarePlan Component:**  **Added Hobbies section under CarePlan for caregivers to select their loved ones’ favourite activities. Introduced more selection options for caregiver’s relationship with patient. Removed heart rate feature as it is no longer relevant for the study.** |
|  | **Enhanced Visual Engagement**: Added visuals and color-coded tabs for headers to improve user interaction and visual appeal. | **Optimized Content**: Enhanced quality with local examples, alternative solutions, and removal of redundancy. | **Clearer Instructions**: Added instructions on the purpose of information icons and improved overall instruction clarity to facilitate navigation. | **Improved Functionality**: Optimized content quality with more recommendations based on feedback, made navigation smoother with automatic redirects on single-choice pages, and added information icons that can be tapped to view definitions. | **In-app Inbox:**  **Added an in-app inbox within the app for users to check on activity and messages from other caregivers and healthcare providers.** |
|  | **User-Centered Tone and Expanded Resources**: Revised solutions for a more empathetic tone and included additional resources based on internal feedback. | **Reduced Text Length**: Shortened content to minimize user fatigue. |  | **User Experience**: Updated Privacy Policy and Technical Support pages, fixed image display issues, improved formatting of bullet points, images, and videos in solutions, and added links to the CareBuddy chatbot and Dementia Singapore helpline at the end of all solutions. |  |
|  | **Optimized Text and Layout**: Improved text formatting, reduced length for readability, and clarified instructions to enhance navigation. | **Increased Visuals**: Added more images and videos for engagement. |  | **Convenience Features**: Added a Bookmark feature for quick access, removed ‘Tap to view more’ buttons for smoother scrolling, improved instructions for the text-to-speech function, and ensured worksheets auto-save and reopen where users left off. |  |
|  |  | **Text-to-Speech**: Implemented text-to-speech functionality for all solutions. |  |  |  |
|  |  | **Highlighted Key Information**: Emphasized important words with bolding or increased font size. |  |  |  |
|  |  | **Expandable Content**: Added ‘View more’ tabs to lengthy solutions for easier navigation. |  |  |  |
| Landing Page | **Enhanced Search Functionality**: Improved accuracy and sensitivity of the 'Search resources/symptoms' feature. | **Reorganized components: Order of the components were rearranged for better grouping on content** | **Improved Tab Organization:** Changed the tab colours of the nine main sections into three rows, each with a distinct colour to represent different categories. | **No Changes** | **Calendar feature: Added in a calendar icon that redirects users to their phone’s calendar function to aid in planning.** |
|  | **Streamlined Content**: Removed the ‘Assessing Your Needs’ section to reduce user fatigue. |  |  |  | **Helpline icon:**  **Added a helpline icon for ease of contacting dementia services** |
|  | **Revised Caregiver Support**: Replaced the ‘Caregiver Distress’ section with three distinct caregiver worksheets: ‘Managing Your Stress’, ‘Caring for Yourself’, and ‘Reflecting on Your Caregiving Journey’. |  |  |  |  |
|  | **Consolidated Dementia Resources**: Merged the ‘Brief Dementia Test’ and ‘Pre-Dementia Screening’ into the ‘Understanding Dementia’ section. |  |  |  |  |
|  | **New Section Added**: Introduced a ‘List of Service Providers’ for additional support. |  |  |  |  |
|  | **Reorganized Landing Page Layout**: Categorized sections on the landing page (top row: dementia symptoms and solutions, middle row: caregiver worksheets, bottom row: miscellaneous dementia information). |  |  |  |  |
|  | **Increased Visibility**: Added icons for Community, Chatbot, and Telemedicine sections at the bottom of the page for better accessibility. |  |  |  |  |
| Behavioural and Physical Symptoms | **Enhanced User Interaction**: Added information icons for users to long-press, providing definitions of specific symptoms for improved understanding. | **Reorganized ADL Solutions:** Modified and restructured ADL solutions, giving each activity its own solution, and separated lengthy solutions into individual symptoms, each with a distinct solution. | **Expanded Content:** Added more symptoms and solutions based on caregiver feedback and encouraging caregivers to use the telemedicine component. | **Mental Health Support:** Provided mental health hotline to solutions addressing mental health issues (e.g., suicidal thoughts) for immediate support**.** |  |
|  |  | **Expanded and Improved Content:** Expanded symptoms and solutions relevant to caregivers, improved phrasing of dementia symptoms to reflect caregiver challenges. |  |  |  |
|  |  | **Enhanced User Experience:** Added more information icons for symptoms needing additional context. |  |  |  |
| Financial Assistance and Planning for your Future | **Section Name Update**: Renamed the section from “Your Financial and Legal Needs” to “Financial Assistance and Planning for Your Future”. | **Section Name Update**: Renamed the section name from ‘Financial Assistance and Planning for your Future’ to ‘Financial Assistance and Future Planning’ to reduce text length | **Updated Financial Assistance Section**: Added definitions for the six ADLs, included information on additional schemes and hotline number with a redirect to the phone keypad. | **Updated Financial Assistance:** Added information about insurance plans for dementia patients under Financial Assistance and fixed previously non-functional hyperlinks. |  |
|  | **Expanded Financial Resources**: Added links to the AIC website for financial schemes and included a link to the My Legacy website in all ‘Planning for Your Future’ solutions. |  | **Expanded Resources**: Included an external link to the Care Services Recommender and added a new tab under Future Planning directing users to Dementia Service Providers. |  |  |
| Understanding Dementia | **Enhanced Solution Structure:** Added four selectable sub-sections before solutions: ‘Stages of Dementia’, ‘Journeying through Dementia’, ‘Reducing the Risk of Dementia’, and ‘Assessing Your Risk of Dementia’. | **Enhanced Dementia Information:** Added a new ‘Different Types of Dementia’ category with details on the four most common types and reorganized the ‘Stages of Dementia’ into three sub-sections: Mild (Early Stage), Moderate (Middle Stage), and Severe (Late Stage). | **New Section Added**: Introduced a ‘Young-onset Dementia’ section under ‘Different Types of Dementia’. | **Updated Dementia Content**: Removed the Pre-dementia Screening Test based on feedback and updated symptoms for each stage of dementia using the ABCD format for better readability. |  |
|  |  |  |  | **New Sections and Resources**: Added a ‘Myths of Dementia’ sub-section and included links to relevant resources (e.g., dementia service providers, financial assistance, future planning) within each Stage of Dementia. |  |
|  | **Improved Image Quality:** Enhanced the clarity of images for better visual understanding. |  | **Test Modifications**: Removed pre-selected default answers for the Pre-dementia Screening Test and Brief Dementia Test. | **Improved Test and Guidance**: Enhanced color coding for the Brief Dementia Test and provided advice to consult a doctor for diagnosis, with a link to Dementia Service Providers if the test result indicates potential dementia. |  |
| List of Service Providers | **Added Disclaimer**: Included a disclaimer with the last updated date for transparency. | **Enhanced Navigation and Filtering**: Added a ‘Deselect All’ button in the filter function and a ‘Home’ button to all pages. | **Clarified Terminology**: Spelled out ‘ACP Providers’ as ‘Advance Care Plan Providers’ to reduce user confusion. |  | **Content Optimization:**  **Updated on location filter tags and website links for some service providers.** |
|  | **Organized Service Providers**: Categorized service providers into three main groups: Dementia Service Providers, Mental Health Providers, and ACP Providers. | **Updated Service Provider Information**: Expanded service provider lists with more comprehensive data from collaborators, including operating hours, contact details, and website links. Removed the service scope filter and added sub-categories under Dementia and Mental Health Providers with definitions for each service scope. | **Enhanced Mental Health Section**: Added information on WhatsApp support groups under ‘Mental Health Helplines’ and fixed formatting issues within ‘Mental Health Providers’. | **Enhanced Filter Function:** The filter now trims results to show only relevant service providers instead of rearranging the full list. Added a ‘Weekend Daycare’ service scope filter under ‘Centre-based Services’ and included more providers under ‘Mental Health Helpline’. |  |
|  | **Service Provider Filter**: Implemented a filter system to search service providers by region, postal code, service scope, and mode of delivery. | **Improved Service Provider Categorization**: Replaced the online/in-person filter with sub-categories under ACP Providers, clarifying that services are intended for both dementia patients and caregivers. |  |  |  |
|  | **Address Updates**: Updated missing addresses for service providers to ensure accuracy. |  |  |  |  |
| Coping with your Grief | **Grief Information and Solutions Reorganization**: Separated content on pre-loss and post-loss grief into two sub-sections and divided grief knowledge from grief solutions into distinct tabs for better clarity. |  | **Text Optimization:** Added ‘View more’ options under bullet points to reduce overall text length. | **Highlighted Mental Health Support:** Bolded and increased the font size for the mental health hotline information in solutions to emphasize its importance. |  |
| Caregiver Worksheets | **Enhanced Navigation**: Improved navigation with clearer arrow buttons, a navigation bar, and a ‘Back’ arrow button for easier movement through the app. | **Enhanced Instruction Clarity**: Improved clarity by breaking long text into paragraphs and bolding key information. | **Enhanced Caregiver Worksheets**: Improved colour coding for selectable options, ensured font colour consistency, and added a link to the List of Service Providers to encourage caregivers to seek help. Replaced the text box with a dropdown menu reflecting selected options in ‘Causes of Stress’. | **Enhanced Features and Support**: Added a voice recording option to all open-ended questions and tangible solutions to help caregivers manage stress. Improved the ‘Journaling’ component for seven-day entries and added more suggestions in ‘Connecting with Others’. |  |
|  | **Fixed Content Save Feature**: Resolved issues with the “Load Previous Content” feature, allowing caregivers to save their answers and review them on future visits. | **Added Support Information**: Included details on where to get support within the CareBuddy app under ‘Causes of Stress’. |  | **Content and Structure Updates**: Introduced a separate tab for Goal Setting under ‘Physical Wellness’ and removed the Downloadable Worksheets tab, now integrated into their respective components. Standardized language and format for all trackers and added information icons for tabs without additional pages. |  |
|  |  |  |  | **Improved Personalization**: Renamed ‘Personal Profile’ to ‘My Favourites’, simplifying questions on caregivers’ favourite activities, and removed the 5-item selection cap in ‘My Caregiver Journey’. |  |
|  |  |  |  | **Added Resources**: Included more visuals to guide caregivers and added a link to Dementia Service Providers for those without help. |  |
| Chatbot | **Enhanced Chatbot Functionality**: Improved the accuracy and intelligence of the chatbot. | **Enhanced Performance:** Improved accuracy and processing speed. | **Improved Sensitivity and Accuracy:** Enhanced the use of layman’s terms and improved overall accuracy. | **Enhanced Accuracy:** Improved overall accuracy throughout the app. | **Enhanced accuracy:**  Improved overall accuracy through the integration of ChatGPT functions. |
|  | **Rebranded Chatbot**: Updated the chatbot name from ‘DUKE’ to ‘CareBuddy’. |  |  |  | **Added Helpline: Included helpline to Dementia Singapore if user requests for it or if the question cannot be answered.** |
|  | **Navigation Improvement**: Added a ‘Back’ button in the external browser window, allowing users to return to the chatbot after clicking on an external link. |  |  |  |  |
|  | **Expanded Recommendations**: Updated the chatbot’s recommendations to include a broader range of services. |  |  |  |  |
| Telemedicine | **Third-Party App Interaction:** Added an explanation informing users that exiting CareBuddy will transition them to a third-party app, and any data will not be carried over. | **Added Disclaimer:** Included a note that services are not free-of-charge. | **No Change** | **No Change** | **Expanded Options:**  Added a new telemedicine provider, Doctor Anywhere, as an additional option for users to consider. |
| Community | **Not available for testing** | **Not available for testing** | **Not available for testing** | **Component Optimization:**  Restricted the ‘delete post’ feature to moderators only and introduced a Moderator site for monitoring and regulating posts. Fixed the ‘like’ feature for user posts and added options to sort posts by alphabetical order, recency, and number of comments. | **Component optimization:**  Fixed the issue where image links cannot be loaded or embedded into a post. |
|  |  |  |  |  | **Notification system:**  Added notifications system where caregivers will be notified whenever there is a new comment to a post, a new pinned post, or when any new post is created. |

**Figure S1: Screenshots of user interface for CareBuddy app**


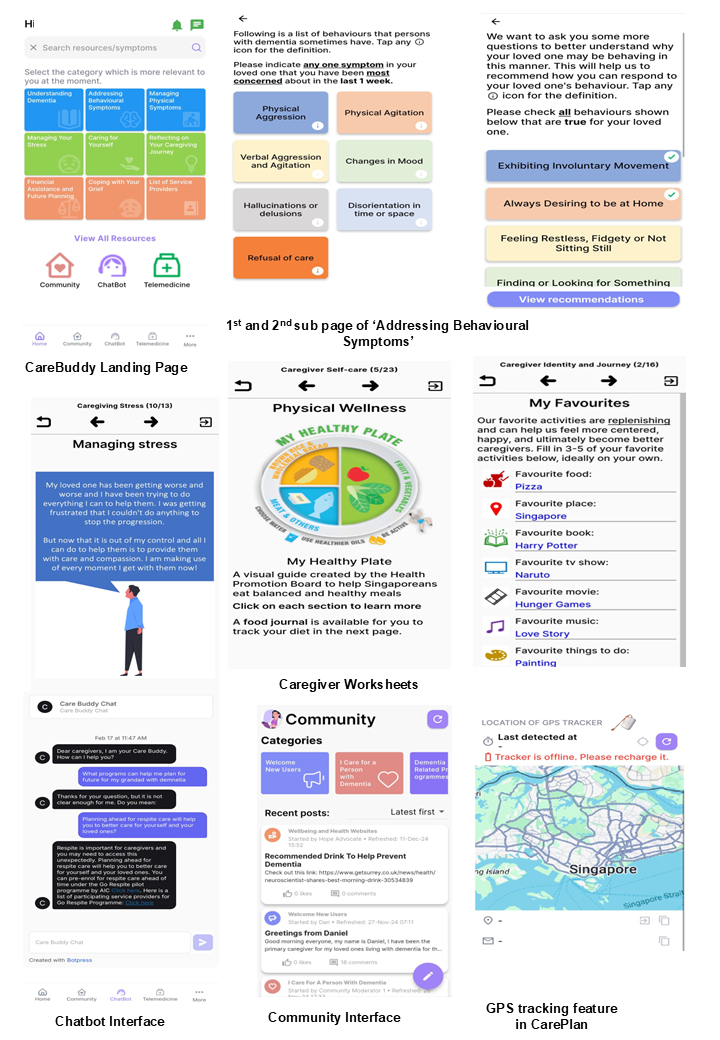

Supplement: Multimedia Appendix 1 [file aging-v8-e78759-s001.docx]
