# Supplementary material for: Supporting Dementia Caregiving With a Mobile Care Ecosystem: Development and Mixed Methods Study
Source: JMIR Aging. 2025 Dec 30;8:e78759. doi: 10.2196/78759 (PMC12810113; doi:10.2196/78759)
Supplement: Multimedia Appendix 2 [file aging-v8-e78759-s002.docx]

**Multimedia appendix 3: Qualitative analysis with illustrative quotes**

| **Dimensions** | **Items** | **Illustrative quotes** |
| --- | --- | --- |
| **Information and content** | Quality of information | *Based on the three different broad categories I have clicked, I think so far, I'm quite impressed. I think that it gives me a quick glimpse of you know why my loved one may exhibit the some behaviour. And then you don't just leave me hanging, you also give me certain solution to work towards.* Phase 1, Participant 16, Health care provider (HCP)  *Because recently also I was invited to test some other app but that one is very, very basic only. .. This one very comprehensive, so I think it's very useful those information. Instead of googling I can go in and look here*. Phase 2, Participant 1, Caregiver |
|  | Quantity of information | *I think it's good - not too long because you also don’t have like two hours. Yeah, it's quite straightforward. And then a I realized they got a summary, quite good. ..The summary is good.*  Phase 1, Participant 17, Caregiver  *I must agree the information there for understanding the dementia behaviours and the physical symptoms are all there. Which I can also find online but, but you have categorized it in such a way that's easy to use.* Phase 2, Participant 10, Caregiver |
|  | Visual information | *Yeah, more pictorial. The first impression I feel because if I see it, I don't know what this is unless I unless I click 1 by 1. Just to be honest***."** Phase 1, Participant 1, Caregiver |
|  | Credibility | *I think it covers quite a bit because you are drawing from AIC (a credible source*). Phase 1, Participant 18, HCP  *Actually, it's impressive of the tips because what I say is good is because it's very practical approach. In fact, all these tips is what we use on our daily routine you see. So, I find that, you know, it works. I find that the tips are very practical, it's good*. Phase 1, Participant 06, HCP |
| **Usability and functionality** | Ease of use & navigation | *It is pretty much comprehensive and easy to navigate. These are the common things that most of them (caregivers) will be able to look at.* Phase 1, Participant 09, HCP |
| **Aesthetics and design** | Layout | *Because I think it's (layout) a bit cluttered and as mentioned, I think (font) is a bit small. Caregivers will appreciate something that's bigger. But I think the picture that accompanies the wording is quite relevant.* Phase 1, Participant 13, HCP |
|  | Graphics | *This is quite interesting because it comes with a lot of pictures.* Phase 1, Participant 09, HCP |
|  | Visual appeal | *I like that you colour coded into the broad category so that, I can instinctively try to find a common theme and then go to it directly. So yeah, I thought that was very nice*. Phase 1, Participant 16, HCP |
| **Engagement** | Interesting | *There's one part which is actually we can actually message fellow caregivers and you can actually read the response from there. Yeah, yeah, because we different stages, right? But you, you can learn from other people that's actually more progressive already, because there are some caregivers who are 16 years of caring.* Phase 2, Participant 07, Caregiver |
|  | Appropriateness | *I like that you all provide the empathetic statement at the start. I think it provides a personal touch to this app…I like that there was emphasis on how we can still interact with persons with dementia at the late stage, because I think in my line of work, this is often a question that many caregivers have*. Phase 1, Participant 13, HCP  *And the based on the solution I see most of them are pretty practical. Yeah, and it's not textbook answer. It can work in real time.."* Phase 1, Participant 1, HCP  *In the beginning I was (checking) almost every day. Not only every day like different times of the day.* Phase 2, Participant 01, Caregiver |
| **Feedback on interactive components** | Community social support | *I think the moderator did quite good job in like throwing questions like what the activities are you do and then I shared. The last one he asked is what do you do at the social club? It's good to share my knowledge. So, I think that is one of the most useful one (component), the one that I go to a lot.* Phase 2, Participant 01, Caregiver |
|  | Chat bot | *At least there are some immediate answers. .. at least, like, you know, you have something to click and that finally you got your answer."* Phase 1, Participant 14, Caregiver  *I think maybe the CareBuddy chat can be more intuitive and more sensitive. And the especially I think am in terms of crisis cases, like if the caregiver talks about suicide, I think it has to be more sensitive to provide the support.* Phase 1, Participant 13, HCP |
|  | Worksheets | *Sometimes you don't really talk to people, but when you when you have this sort of question in the worksheet and this could be the common thing that your fellow caregiver are going through as well. So, you know from there. You read about it and then you write your own thing. It is better.* Phase 2, Participant 07, Caregiver  *Yes, I do. I learned to say, I mean now I understand myself, better. Meaning I sometimes stress is not just external, essentially internal. Cause of my personal stress and then plus my the caregiving stress is all mixed up. So, I just get help. I did get some very good advise on how to manage.* Phase 2, Participant 06, Caregiver |
|  | Listing and directory | *I saw the list and I found the ones (service providers) that are nearer to my house. And of course, I'm very happy with the phone numbers. Because very important to have phone numbers I can call immediately*. Phase 2, Participant 06, Caregiver |
|  | GPS tracker | *The main thing is the app, the tracker. That's the main thing.. I like the GPS. But the only problem with the GPS is it is a delayed motion. If you can make it faster then they're very good. Helpful for me as a caregiver actually.* Phase 2, Participant 02, Caregiver |
| **Perceived usefulness** | Improved knowledge | *I think the app itself, gives you a lot of information. If the doctor had given me that when he (my father) was first diagnosed, then I will have more knowledge.* Phase 2, Participant 03, Caregiver |
|  | Supporting caregivers | *I think it's very helpful to care partner like me. When I need help, I know this app is around. When first diagnosed, I was not too sure what to do. Totally lost, feel so scary, sad…upset, worrying, yeah. So, it's like, you know, very helpless, do not know where to go.* Phase 1, Participant 14, Caregiver  *It (CareBuddy app) helped me to understand them better and also notice any new symptoms. This is what I can see that…In fact, just by calling her (aunt’s) name two or three times, she (aunt) got like agitated.  But, in fact we don't ask her to do anything. This is one new behaviour that I noticed. But then (after using the app) I understand how to approach (her). We cannot be calling her from far, so it's better to have like eye-to-eye contact and she may respond better. So, it's (solutions) are quite helpful.* Phase 2, Participant 04, Caregiver |
| **Overall impression** | Comprehensive and resourceful | *Don’t wait five years. Yeah. Just launch it. We need this kind of resources. I mean, things like this, this is really very well done. Why wait five years* Phase 1, Participant 11, Caregiver  *I am very excited for this app to be launched because right now in the market don’t think there is really an app which covers so much information* Phase 1, Participant 16, HCP  *Then actually for people like us, we don’t have time to look up so many apps. One that actually have everything (is good). We don’t need to have a Cara app... Actually, the information (in this app) is good enough. .. I like this CareBuddy, very straightforward.* Phase 2, Participant 02, Caregiver |
|  | Recommend to other caregivers | *Yes. I just realised that so many people around me have the same issue. I was talking to my colleagues all that and I realised some of them might actually have same issues. Yeah, I was like I want to show this to them*. Phase 2, Participant 09, Caregiver |
